# Optimal substrate composition (C, N, and trace metals) for liquid culture of *Akanthomyces attenuatus* JEF 147 isolate

**DOI:** 10.3389/ffunb.2026.1818832

**Published:** 2026-06-30

**Authors:** Muhammad Farooq, Dan-Dan Zhao, Waqar Ahmad, Zakirullah Khan, Rahmatullah Jan, Bo-Seong Seo, Yonghwa Lee, Kyung-Min Kim

**Affiliations:** 1 Department of Agricultural Biology, College of Agriculture & Life Sciences, Jeonbuk National University, Jeonju, Republic of Korea; 2 Department of Applied Biosciences, Graduate School, Kyungpook National University, Daegu, Republic of Korea; 3 Crop Environment Research Division, National Institute of Crop and Food Science, Rural Development Administration, Wanju, Republic of Korea; 4 Department of Agriculture Convergence Technology, Jeonbuk National University, Jeonju, Republic of Korea; 5 Coastal Agriculture Research Institute, Kyungpook National University, Daegu, Republic of Korea

**Keywords:** *Akanthomyces attenuates* JEF 147, blastospores, potato starch, tryptone, zinc sulfate

## Abstract

*Akanthomyces attenuatus* JEF 147 is a promising entomopathogenic fungus with notable acaricidal activity against spider mites. Controlled submerged cultivation with defined nutritional sources enables efficient mycelial and blastospore production while revealing vital features of fungal metabolism and adaptability. This study aimed to optimize liquid culture conditions for the production of *A*. *attenuatus* JEF 147, focusing on various carbon sources (fructose, glucose, maltose, sucrose, and potato starch), nitrogen sources (peptone, tryptone and yeast extract), and trace metals, (copper(II) sulfate, iron(II) sulfate, magnesium sulfate, manganese(II) sulfate, and zinc sulfate). Among the carbon sources tested, potato starch yielded the blastospores count 32 × 10^7^ after 7 days of culture. The nitrogen source, tryptone, produced the highest number of blastospores, up to 42 × 10^7^, representing a 31.3% increase over potato starch. Meanwhile, optimizing the carbon-to-nitrogen (C:N) ratio to 0.5:1 further increased blastospores production to 67 × 10^7^, a 109.4% increase compared with potato starch and a 59.5% over tryptone alone. Zinc sulfate (ZnSO_4_) as a trace metal resulted in a blastospores count of 37 × 10^7^, indicating a 11.9% decrease compared with tryptone and a 44.8% decrease compared with the 0.5:1 C:N condition. These results suggest that a C:N ratio of 0.5:1 maximizes blastospore production; furthermore, the addition of trace metals enhances the size and morphology of *A. attenuatus* blastospores but reduces total blastospore production. This study highlights the importance of optimizing culture conditions for the production of *A*. *attenuatus* JEF 147 spores, benefiting both research and industrial applications.

## 1 Introduction

Recently, the potential use of entomopathogenic fungi in biological control strategies and bioinsecticide production has gained substantial attention (Kumar et al., 2019; Jiang and Wang, 2023). Subsequently, the commercial presence of entomopathogenic fungi and overall use have expanded; however, differences in national registration systems make global tracking difficult. Nonetheless, the established use of products such as Botani Gard, Biofact, Naturalist L, Broadband, Chongcheaesak, Velififer and Green Muscle has been validated (Kim et al., 2020). Previously, many researchers believed that fungal bioinsecticides could replace chemical insecticides; thus, subsequent research has focused on improving the associated efficacy and application strategies (Bravo and Soberón, 2023). However, while fungal insecticides have been studied for several years, overall developmental innovation remains limited (Lacey et al., 2015).

Blastospores are yeast-like vegetative cells, similar to the hyphal bodies usually produced during infection by entomopathogenic fungi within the haemocoel of arthropods (Dietsch et al., 2021; Mascarin et al., 2021) and have been considered as a promising propagule for use in biological control programs (Iwanicki et al., 2020; Mascarin et al., 2023). Blastospores of entomopathogenic fungi can rapidly kill aphids and whiteflies (Huang et al., 2020) and can be efficiently produced in large quantities in liquid media in a short time and using limited space (Behle et al., 2022).

Entomopathogenic fungi exhibit differential growth across various media; however, the most commonly used techniques for fungal spore production are either surface culture on a solid substrate (e.g., wheat bran, millet, or rice) or submerged liquid culture (Feng et al., 1994). Earlier studies demonstrated that blastospore production in the entomopathogenic fungus *Beauveria bassiana* was enhanced by appropriate carbon and nitrogen sources under rotatory incubator conditions (200 rpm) (Pham et al., 2009). A recent study also demonstrated that submerged liquid media containing C:N sources enhanced blastospore production, made the blastospores shelf-stable, and produced more virulent propagules for useful pest biocontrol programs (Lima et al., 2024). Furthermore, some fungi, specifically saprophytes, can digest a wide variety of nitrogen sources, of which some are favorably absorbed with higher proficiency than others. Different nitrogen sources activate distinct metabolic pathways during digestion (Marzluf and Reviews, 1997) resulting in diverse metabolic byproducts that alter cytoplasmic and membrane fatty acid composition, and cellular antioxidant capacity (Torija et al., 2003; El-Sheekh et al., 2004; Ismaiel, 2016).

Currently, these fungal strains are mainly used in combination with chemical insecticides to address issues such as insecticide resistance and harmful residue buildup. This particular strain can infect and kill specific insect pests, making the strain a promising agent for environmentally friendly pest management. However, understanding the optimal growth conditions for this fungal strain is critical, particularly in submerged liquid culture, which is ideal for large-scale spore production due to the associated scalability and precise environmental control. In submerged culture, various carbon, nitrogen and trace metal sources can significantly influence fungal growth and spore production. Therefore, this study aimed to identify optimal combinations of carbon, nitrogen and trace metals nutrient sources to enhance spore yield and improve the quality of blastospores produced by *Akanthomyces attenuatus* JEF 147.

## 2 Materials and methods

### 2.1 Culture and utilization of *Akanthomyces attenuatus* JEF 147

**Figure 1:**
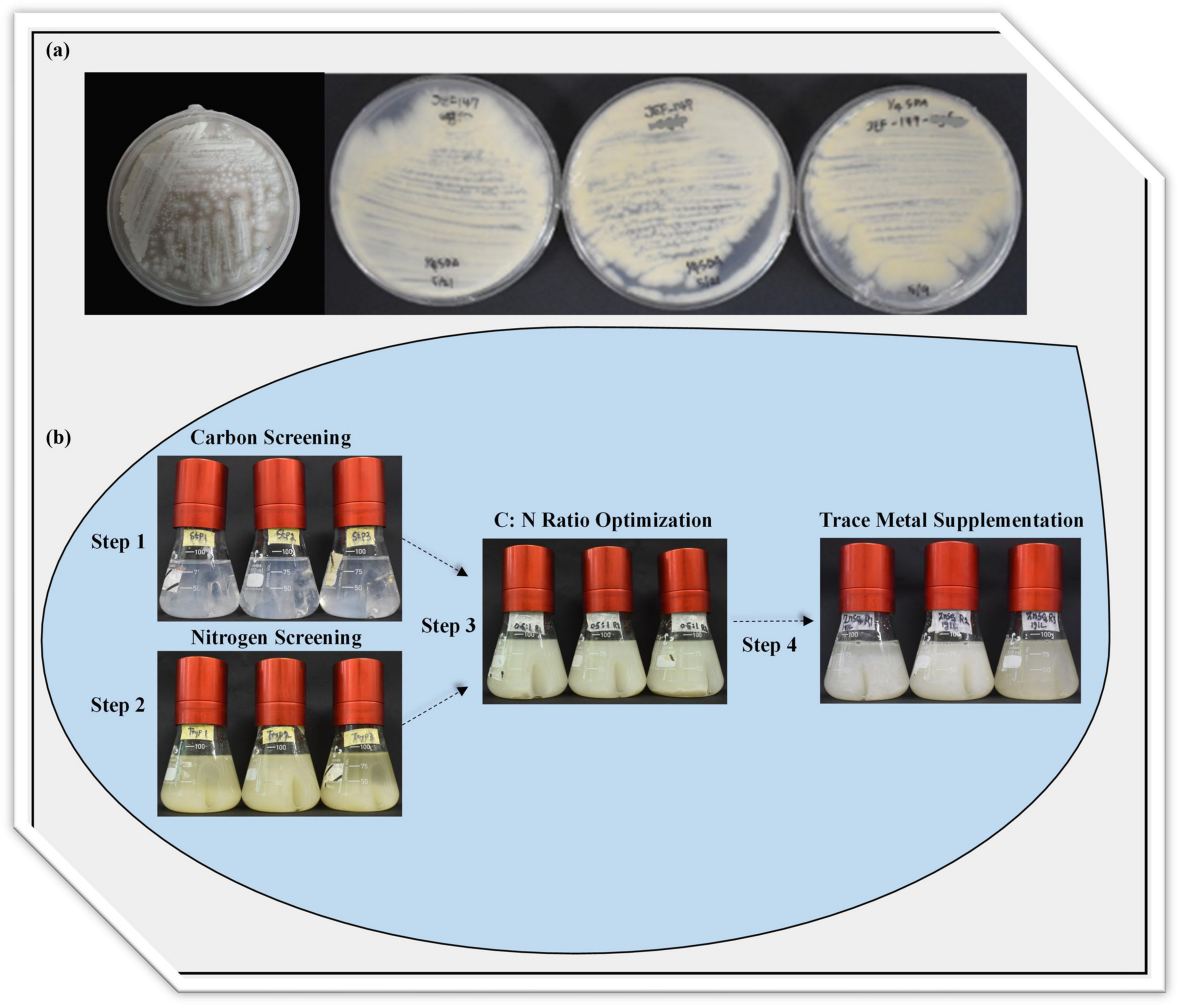
Experimental workflow for optimization of liquid culture conditions for blastospores production of *Akanthomyces attenuatus* JEF 147 **(A, B)**. Step 1 and step 2 evaluate the effects of different carbon and nitrogen sources individually on blastospores production of *A attenuatus* JEF 147. In step 3, the selected carbon source (potato starch) and nitrogen source (tryptone) were combined at different mixing ratios to determine the optimal carbon-to-nitrogen (C:N) ratio for blastospore production. In step 4, after determining the optimal C:N ratio, various trace metals were supplemented into the medium to further assess their effects on blastospore production of *A. attenuatus* JEF 147.

This study used the *Akanthomyces attenuatus* JEF 147 isolate provided by the Insect Microbiology & Biotechnology Laboratory (IMBL) at the College of Agriculture & Life Sciences, Jeonbuk National University, Jeonju, Korea. Morphologically, this isolate appears as a fluffy, white fungus that generally grows after 48 hours on ¼-strength Sabouraud dextrose agar (SDA) medium (**Figure 1A**). This entomopathogenic fungus has unique characteristics and was initially used in the IMBL to kill spider mites. However, this study used different carbon, nitrogen, and trace-metal sources to optimize the growth rate of *Akanthomyces attenuatus* JEF 147 blastospores (**Figure 1B**).

### 2.2 Growth rate of *Akanthomyces attenuatus* JEF 147 under different carbon sources

This study used various carbon sources, including fructose, glucose, maltose, sucrose and potato starch, each at 1% (**Table 1**). To inoculate *Akanthomyces attenuatus* JEF 147 into these carbon sources, the *Akanthomyces attenuatus* JEF 147 fungal strain was initially cultured on ¼-strength SDA medium for 7 days (**Supplementary Figure 1**). Then, the conidia of *Akanthomyces attenuatus* JEF 147 were suspended in 15 mL of 0.03% siloxane solution (Silwet, Farm-Hanong Inc., Nonsan, Korea) and adjusted to 1 × 10^7^ conidia/mL to prepare the conidial suspension. Flasks containing the carbon sources were autoclaved and 1 mL of *Akanthomyces attenuatus* JEF 147 was inoculated into each replicate. The flasks were then placed in a shaking incubator at 170 rpm and 25 °C for 7 days. Spore production and conidial growth were evaluated daily under a microscope. Spore counts were determined using hemocytometer under a light microscope following standard dilution formula and counting procedure. This method was applied consistently across all treatments (S2).

**Table 1:** Preparation of a 1% carbon source solution in distilled water.

Carbon sources	Fructose	Glucose	Maltose	Sucrose	Potato starch
Composition	1 g/100 mL	1 g/100 mL	1 g/100 mL	1 g/100 mL	1 g/100 mL


$$\text{Blastospores }{\text{mL}}^{-1}=\text{Average cell count}\times \text{dilution factor count}\times 4\times 10^{6}$$

### 2.3 Utilization of nitrogen sources for growth and blastospores production of *Akanthomyces attenuatus* JEF 147

The current study used three different nitrogen sources, peptone, tryptone and yeast extract, each as a 1% solution (**Table 2**). A 15 mL conidial solution of *Akanthomyces attenuatus* JEF 147 was prepared in 0.03% siloxane solution (Silwet FarmHanong Inc., Nonsan, Korea). Then, 1 mL of suspension of *Akanthomyces attenuatus* JEF 147 was added to each nitrogen-source replicate. The flasks were placed in a shaking incubator at 170 rpm and 25 °C for 7 days (**Supplementary Figure 3**). The growth rate and blastospore production of *Akanthomyces attenuatus* JEF 147 were assessed daily under a microscope.

**Table 2:** Preparation of a 1% nitrogen source solution in distilled water.

Nitrogen sources	Peptone	Tryptone	Yeast extract
Composition	1 g/100 mL	1 g/100 mL	1 g/100 mL

### 2.4 Carbon and nitrogen source combination with different mixture ratios

We prepared various combinations of carbon and nitrogen sources using a statistical design matrix. Potato starch was selected as the carbon source and tryptone as the nitrogen source (**Table 3**). The *Akanthomyces attenuatus* JEF 147 strain was inoculated with different C:N ratios (1:1, 2:1, 0.5:1, 1:2, and 1:0.5) and the resulting growth rates were evaluated (**Supplementary Figure 4**).

**Table 3:** A 1% solution of carbon and nitrogen sources in different mixture ratios.

Mixture ratio	Carbon source (potato starch)	Nitrogen source (tryptone)	1% solution composition from different ratio mixtures (100 mL) C:N
1:1	1	1	500 mg + 500 mg
2:1	2	1	667 mg + 333 mg
0.5:1	0.5	1	333 mg + 667 mg
1:2	1	2	333 mg + 667 mg
1:0.5	1	0.5	667 mg + 333 mg

### 2.5 Inoculation of different trace metals into a combined carbon and nitrogen source

To optimize the best mixture composition for the growth of *Akanthomyces attenuatus* JEF 147, we tested various trace metals, including copper (II) sulfate (CuSO_4_.5H_2_O), iron(II) sulfate (FeSO_4_.7H_2_O), magnesium sulfate (MgSO_4_.7H_2_O), manganese(II) sulfate (MnSO_4_.H_2_O) and zinc sulfate (ZnSO_4_). These trace metals were combined in different compositions (**Table 4**), with carbon (potato starch) and nitrogen (tryptone). Based on the carbon-to-nitrogen ratio, we selected a C:N ratio of 0.5:1, added 1 mL of the trace-metal stock solution, and evaluated the growth rate of *Akanthomyces attenuatus* JEF 147 (**Supplementary Figure 5**).

**Table 4:** Trace metals, formulas, and the associated compositions.

Trace metals	Formula	Composition
Copper(II) sulfate	CuSO_4_.5H_2_O	1 g/L
Iron(II) sulfate	FeSO_4_.7H_2_O	0.5 g/L
Magnesium sulfate	MgSO_4_.7H_2_O	2.5 g/L
Manganese(II) sulfate	MnSO_4_.H_2_O	0.5 g/L
Zinc sulfate	ZnSO_4_	1 g/L

### 2.6 Statistical analysis

The experiment comprised four treatments, each with triplicates. The experiment was repeated three times and the combined data are presented as the mean ± standard deviation. Statistical comparisons were performed using *post hoc* Tukey tests in a one-way analysis of variance (ANOVA). Different letters on the vertical bars indicate statistically significant differences at ^c^*p* < 0.05, ^b^*p* < 0.01, ^a^*p* < 0.001.

## 3 Results

### 3.1 Effect of carbon sources on growth and blastospores production *Akanthomyces attenuatus* JEF 147

**Figure 2:**
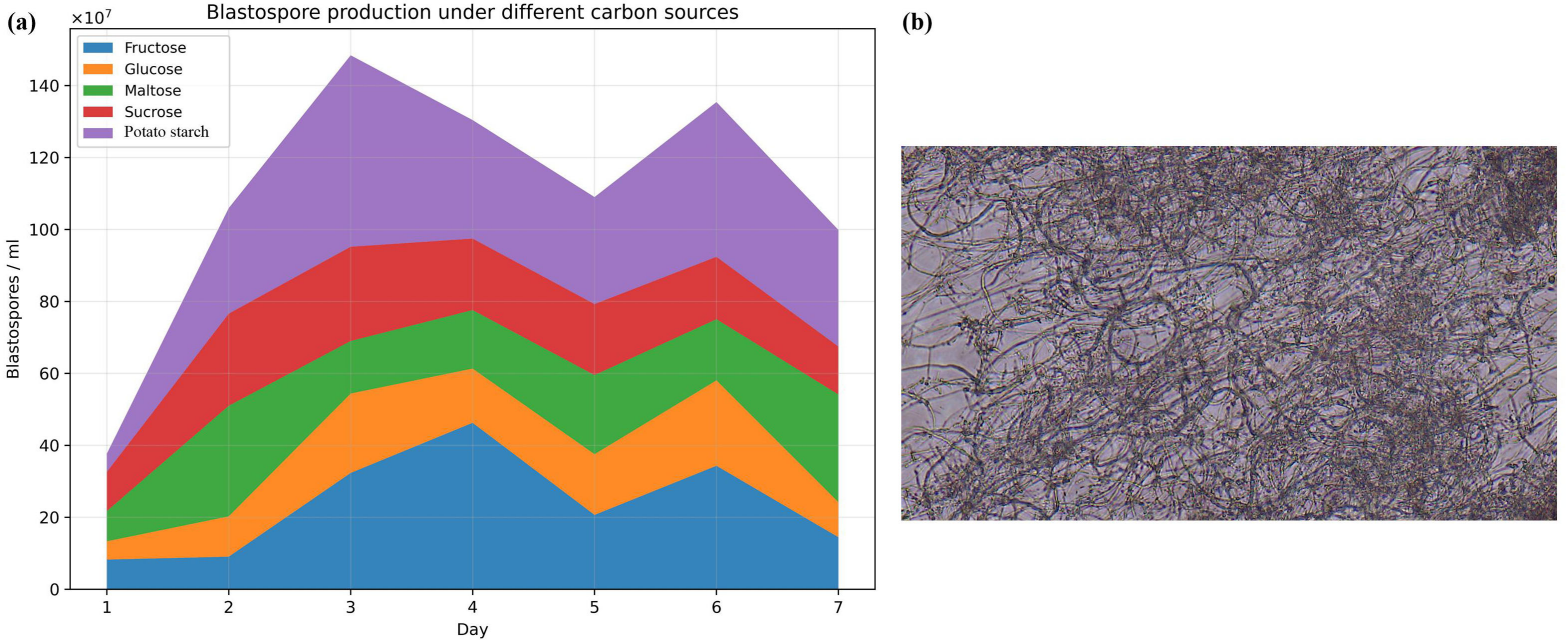
Average spore production (32 × 10^7^ blastospores/mL) on day 7 of incubation in a liquid medium supplemented with 1% potato starch as the carbon source. The statistical analysis indicates a significant difference across treatments (F(4,10) = 6.5; *p* = 0.007), confirming that potato starch significantly enhances blastospore production compared with the other substrates.

The effect of different carbon sources on the growth and blastospores production of *Akanthomyces attenuatus* JEF 147 was evaluated using five carbon sources: fructose, glucose, maltose, sucrose, and potato starch. Among these, potato starch proved to be the most effective, yielding the highest blastospore count (32 × 10^7^) after incubation at 170 rpm for 7 days. In addition to the enhanced blastospore yield, cultures grown with potato starch showed well-developed mycelial structures, indicating healthy, vigorous fungal growth. Maltose also supported blastospore production and mycelial development; however, the performance of the maltose source was lower than that of potato starch. Additionally, the other carbon sources resulted in comparatively lower spore counts and less pronounced mycelial formation. These results show that the choice of carbon source plays a critical role in determining both the quantitative and qualitative aspects of fungal development (**Figures 2A, B**).

### 3.2 Effect of nitrogen source on growth and blastospores production of *Akanthomyces attenuatus* JEF 147

**Figure 3:**
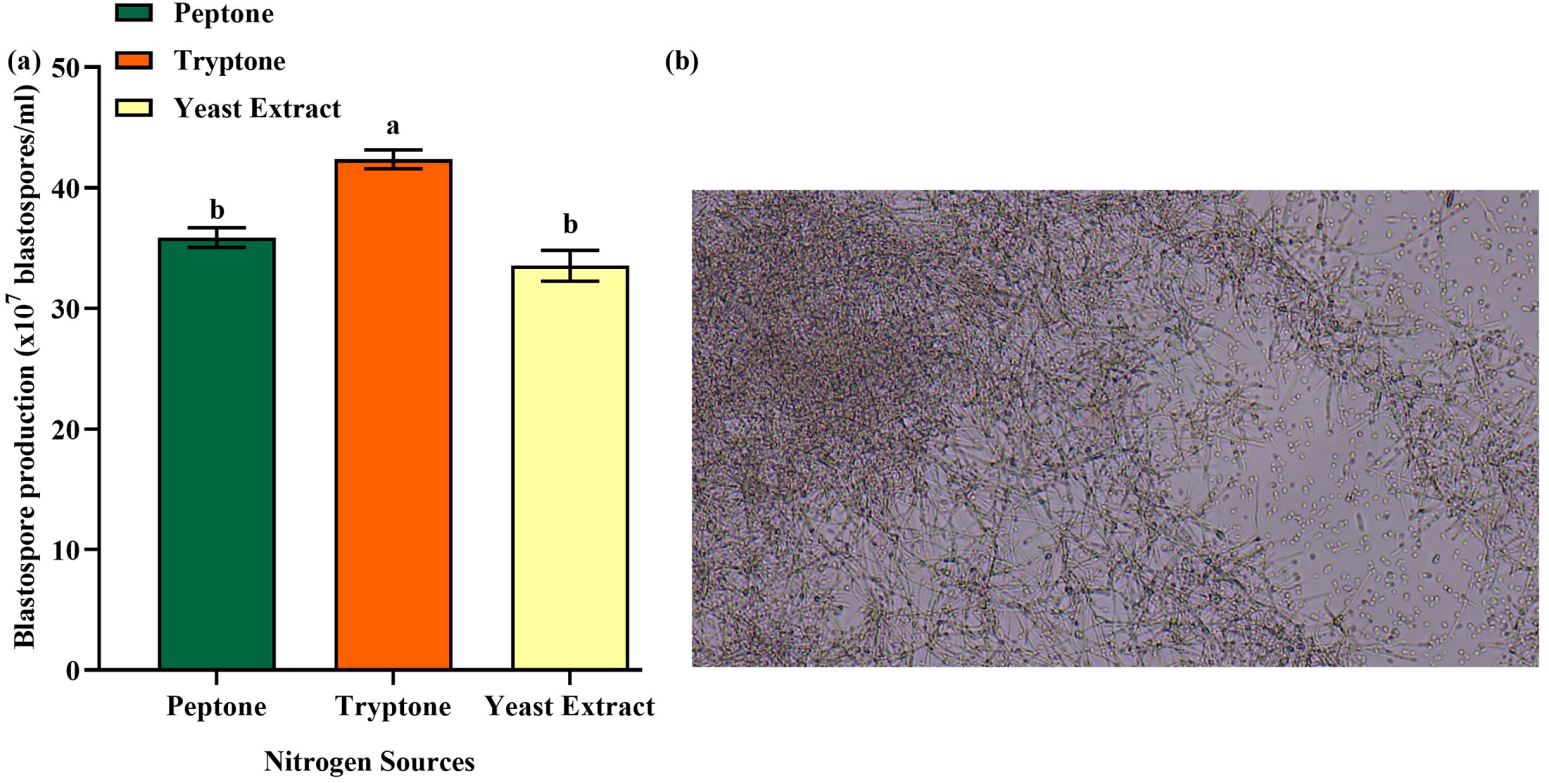
Average spore production (42 × 10^7^ blastospores/mL) on day 7 of incubation in a liquid medium supplemented with 1% tryptone as the nitrogen source. Statistical analysis across treatments indicated significant differences (F(3,5) = 1.79; *p* = 0.264).

To assess the role of nitrogen nutrition, the effects of organic nitrogen sources, including peptone, tryptone, and yeast extract, on fungal growth and sporulation were evaluated. Among these sources, tryptone was identified as the most effective, yielding 42 × 10^7^ blastospores after incubation at 170 rpm for 7 days. This indicated a 31.3% increase in blastospore production compared with cultures grown with potato starch as the carbon source. Cultures supplemented with tryptone showed enhanced conidia accumulation and dense blastospore formation, indicating that tryptone provides readily available nitrogen and essential amino acids that favor fungal metabolisms and reproduction. Peptone and yeast extract also supported growth and blastospores production, but the effects of these sources on blastospores production were comparatively lower than those of tryptone (**Figures 3A,B**).

### 3.3 Effect of combined carbon and nitrogen ratio on blastospores production

**Figure 4:**
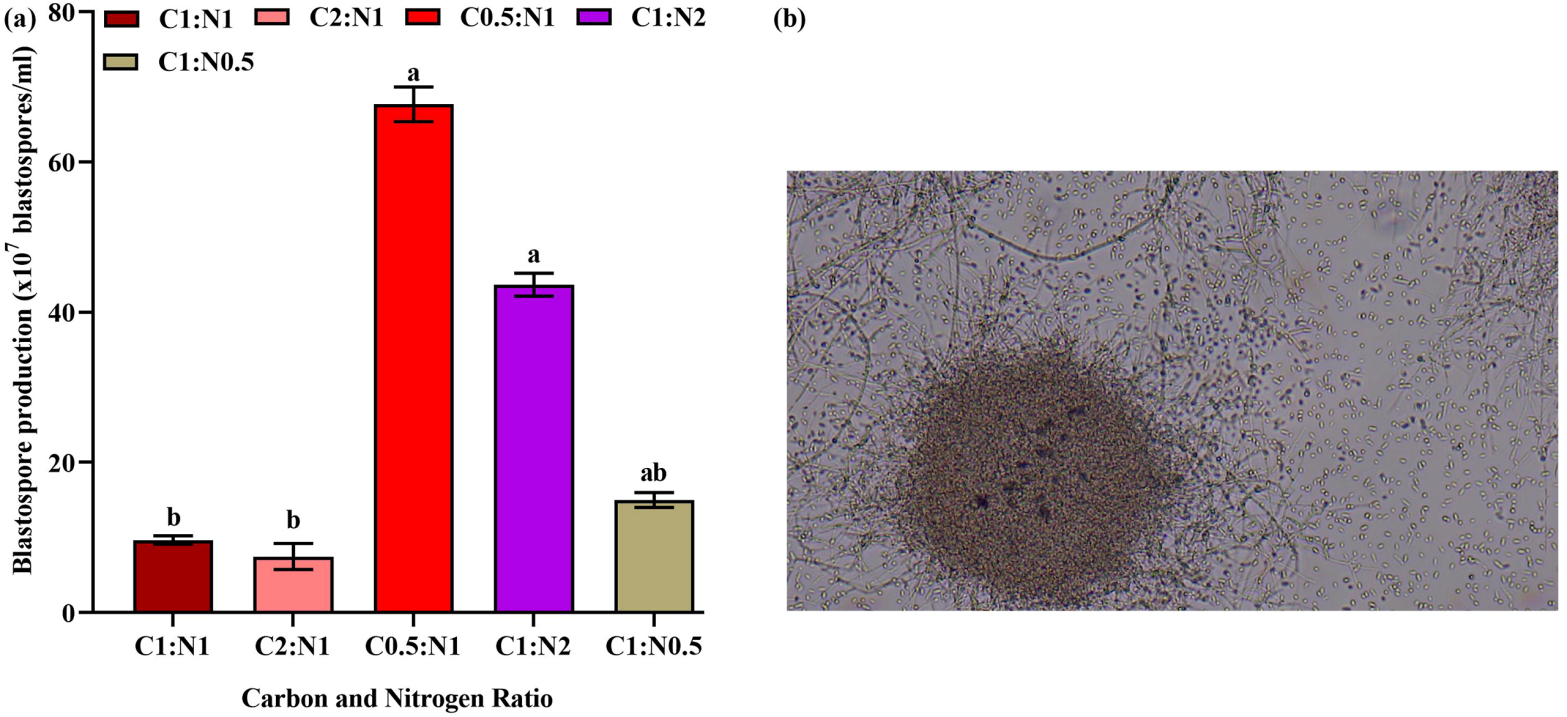
Spore production (67 × 10^7^ blastospores/mL) on day 7 in a liquid medium with a 0.5:1 carbon-to-nitrogen ratio (C:N), using potato starch as the carbon source and tryptone as the nitrogen source, cultured at 27 °C and 170 rpm. A significant effect was observed (F(4,10) = 7.8; *p* = 0.004).

Given the strong individual effects of potato starch and tryptone, further tests were conducted to optimize the carbon-to-nitrogen (C:N) ratio. A C:N ratio of 0.5:1 was selected, with potato starch as the carbon source and tryptone as the nitrogen source. Optimization at this ratio yielded a blastospores count of 67 × 10^7^, representing a 59.5% increase over the tryptone source alone. The optimized C:N ratio not only enhanced spore production but also produced uniform, actively dividing blastospores, suggesting an improved metabolic balance between carbon and nitrogen availability. These observations indicate that appropriate nutrient ratios are essential for maximizing fungal propagative potential (**Figures 4A,B**).

### 3.4 Effect of trace metal sources on blastospore growth and quality

**Figure 5:**
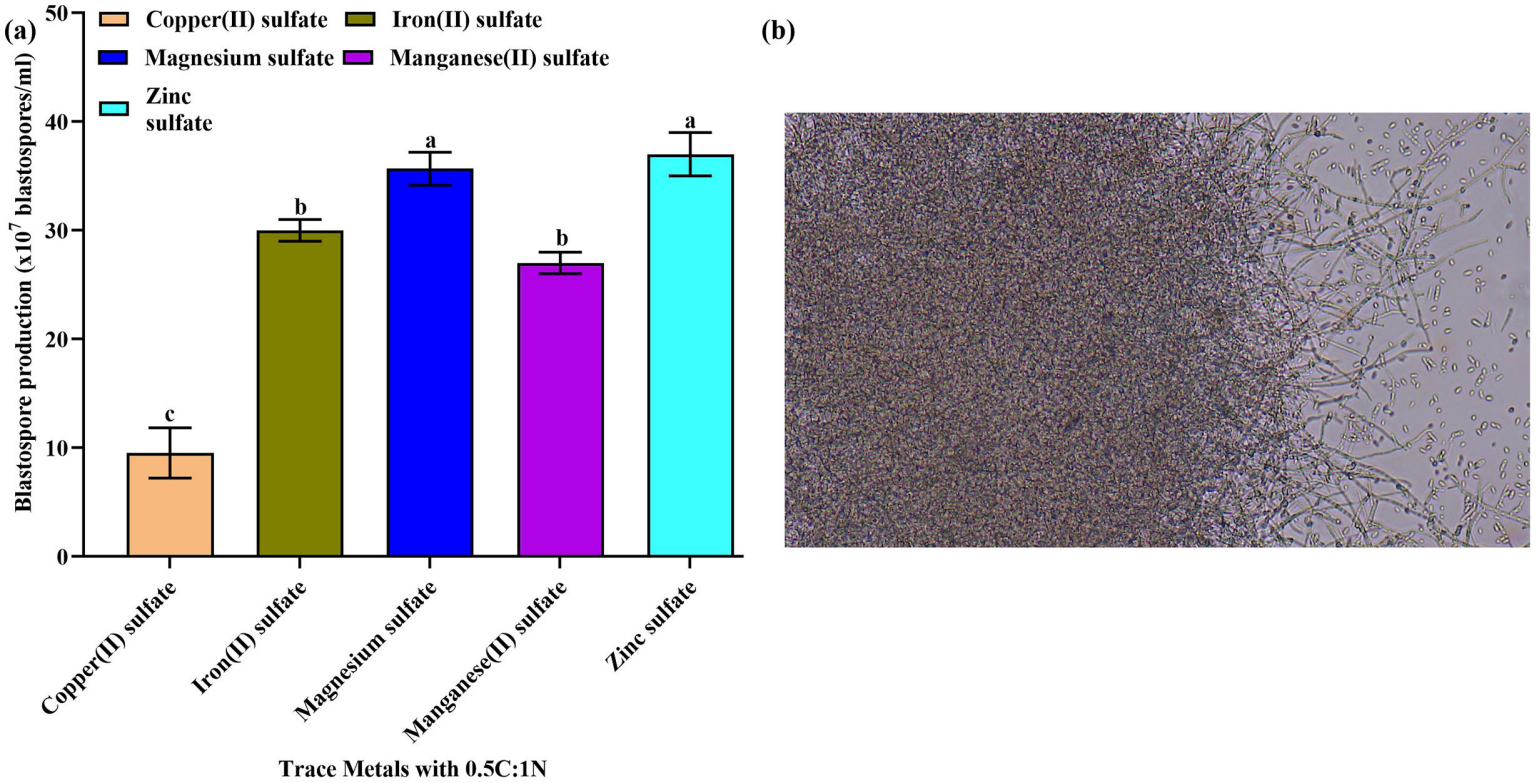
Spore production (37 × 10^7^ blastospores/mL) on day 7 of incubation in a liquid medium with a 0.5:1 carbon to nitrogen ratio (C:N) supplemented with potato starch, tryptone, and zinc sulfate as a trace metal source. The culture was incubated at 25 °C and 170 rpm. Statistical analysis showed no significant effect (F(4,10) = 1.014; *p* = 0.445).

In addition to the carbon and nitrogen sources, the effects of trace metals (CuSO_4_, FeSO_4_, MgSO_4_, MnSO_4_, and ZnSO_4_) were tested on fungal growth and sporulation. Among the trace metals tested, zinc sulfate (ZnSO_4_) had a distinct effect on the appearance of blastospores. Although supplementation with ZnSO_4_ yielded a blastospores count of 37 × 10^7^, reflecting a 11.9% decrease compared with tryptone and a 44.8% reduction compared with the optimized 0.5:1 C:N ratio, this trace metal remarkably enhanced the size, uniformity, and overall morphology of the blastospores. This suggests that while ZnSO_4_ may slightly reduce total spore counts, this trace metal improves blastospore quality, which could be advantageous in specific contexts, such as enhancing cellular quality despite reduced total numbers. These results highlight the importance of optimizing nutrient sources and the associated ratios to maximize blastospore production and improve blastospore quality for potential use in biological control and bioinsecticide production. The 0.5:1 C:N ratio, with ZnSO_4_ supplementation, appears to be the most promising for enhancing the size and morphology of *Akanthomyces attenuatus* JEF 147 blastospores, providing valuable insights for both research and industrial applications (**Figures 5A, B**).

## 4 Discussion

The influence of nutrient composition on growth and blastospore production observed in the current study is consistent with several reports on entomopathogenic fungi, such as *Beauveria*, *Metarhizium*, and *Cordyceps* species. Carbon source complexity has repeatedly been identified as a key determinant of the efficacy of fungal biomass accumulation. For example, studies on *Beauveria bassiana* and *Metarhizium anisopliae* have confirmed that complex carbohydrates, such as starch and other polysaccharide-rich substrates, yield higher blastospore counts than simple sugars, possibly due to sustained carbon availability and reduced catabolite repression (Jackson et al., 1997; Mascarin et al., 2021). These findings closely align with ours, in which the potato starch source outperformed monosaccharides in stimulating mycelial development and sporulation of *Akanthomyces attenuatus* JEF 147 (**Figure 2**).

Tryptone and yeast-derived nitrogen sources are frequently associated with enhanced sporulation due to their balanced amino acid composition and the presence of growth-promoting peptides. In *Isaria fumosorosea*, tryptone-based media markedly increased blastospore production and virulence compared with inorganic nitrogen sources (Pham et al., 2009). Similarly (Gao et al., 2019), reported that organic nitrogen sources supported rapid cell division and higher blastospore viability in *Beauveria brongniartii*. However, in the present study, nitrogen sources such as tryptone, peptone, and yeast extract significantly increased blastospore production compared with carbon sources, with tryptone yielding the highest. These results demonstrate that the balanced amino acid composition of these nitrogen sources might be responsible for enhanced blastospore production and metabolic activity observed in *Akanthomyces attenuatus JEF* 147 (**Figure 3**).

Notably, C:N ratio optimization has been reported to play a vital role in fungal development. Excess carbon generally promotes vegetative growth, whereas nitrogen limitation can restrict sporulation, underscoring the importance of a balanced nutrient ratio. A previous study on *Metarhizium robertsii* reported that intermediate C:N ratios maximize blastospore production by promoting efficient energy use and cellular differentiation (Jaronski and Jackson, 2012). Our observation that a C:N ratio of 0.5:1 resulted in higher blastospore production in *Akanthomyces attenuatus* supports the concept that optimal nutrient balance, rather than absolute nutrient concentration, enhances sporulation efficiency in liquid culture systems (**Figure 4**).

The trace metal effects observed in this study are also consistent with prior research. A previous study reported that zinc is an essential micronutrient that influences enzyme activation, membrane stability, and oxidative stress tolerance in fungi. Indeed, zinc supplementation in *Beauveria bassiana* enhanced conidial quality, stress resistance, and shelf-life stability, even when spore numbers did not increase (Rangel et al., 2015). Similarly (Cui, 2015), demonstrated that Zn^2+^ improved cell wall integrity and blastospore vigor in *Cordyceps militaris*. The increase in blastospore size and improved morphology observed under ZnSO_4_ supplementation in our study suggests a comparable role for zinc in enhancing cellular quality rather than quantity, which may be particularly beneficial for formulation stability and field efficacy.

Collectively, our study is consistent with previous research and advances current knowledge by providing the first detailed nutrient optimization framework for the entomopathogenic fungus *Akanthomyces attenuatus* JEF 147 in liquid medium. In contrast to previous studies that often focus on single nutritional factors, our integrated approach highlights the synergistic effects of carbon type, nitrogen source, C:N ratio, and trace metal supplementation (**Figure 5**). This comprehensive optimization approach strengthens the potential of *Akanthomyces attenuatus* JEF 147 as a scalable and effective biological control agent and provides a solid foundation for fermenter-based mass production studies.

## 5 Conclusion

Optimizing carbon and nitrogen sources and the associated ratios significantly enhances spore production in *Akanthomyces attenuatus* JEF 147. Potato starch, and tryptone were the most effective substrates, and a C:N ratio of 0.5:1 was optimal for both growth and sporulation. Although ZnSO_4_ reduced overall spore counts, this trace metal improved the size and morphology of blastospores, indicating potential for specific applications. These findings provide a basis for optimizing culture conditions to maximize the efficiency and quality of spore production for biological control purposes.

## Data Availability

The raw data supporting the conclusions of this article will be made available by the authors, without undue reservation.
