# Correction: Optimal substrate composition (C, N, and trace metals) for liquid culture of *Akanthomyces attenuatus* JEF 147 isolate

**DOI:** 10.3389/ffunb.2026.1926693

**Published:** 2026-07-28

**Authors:** Muhammad Farooq, Dan-Dan Zhao, Waqar Ahmad, Zakirullah Khan, Rahmatullah Jan, Bo-Seong Seo, Yonghwa Lee, Kyung-Min Kim

**Affiliations:** 1 Department of Agricultural Biology, College of Agriculture & Life Sciences, Jeonbuk National University, Jeonju, Republic of Korea; 2 Department of Applied Biosciences, Graduate School, Kyungpook National University, Daegu, Republic of Korea; 3 Crop Environment Research Division, National Institute of Crop and Food Science, Rural Development Administration, Wanju, Republic of Korea; 4 Department of Agriculture Convergence Technology, Jeonbuk National University, Jeonju, Republic of Korea; 5 Coastal Agriculture Research Institute, Kyungpook National University, Daegu, Republic of Korea

**Keywords:** *Akanthomyces attenuates* JEF 147, blastospores, potato starch, tryptone, zinc sulfate

Authors Dan-Dan Zhao, Bo-Seong Seo, and Yonghwa Lee were erroneously assigned to affiliation 1. This affiliation has now been removed for these authors. 

Affiliation 3 "Crop Environment Research Division, National Institute of Crop and Food Science, Rural Development Administration, Wanju, Republic of Korea" was omitted from the paper. This affiliation has now been added for authors Dan-Dan Zhao, Bo-Seong Seo, and Yonghwa Lee. Subsequent affiliations have been renumbered.

In the **Acknowledgments**, the wrong project number was included and one project was omitted. The **Acknowledgments** initially read: “The work carried out with the support of Cooperative Research Program for Agriculture Science and Technology Development (Project No. RS-2026-25516544) of the Rural Development Administration, Republic of Korea.”

The final **Acknowledgments** now reads:

“The work was carried out with the support of the Cooperative Research Program for Agriculture Science and Technology Development (Project No. RS-2025-02214096), Rural Development Administration, Republic of Korea, and of the Key Country-Focused Technology Research and Development Cooperation Project of Yancheng (Project No. Ycgh2025004).”

The original version of this article has been updated.

