# Supplementary material for: Optimal substrate composition (C, N, and trace metals) for liquid culture of Akanthomyces attenuatus JEF 147 isolate
Source: Front Fungal Biol. 2026 Jun 30;7:1818832. doi: 10.3389/ffunb.2026.1818832 (PMC13364918; doi:10.3389/ffunb.2026.1818832)

Phenotypic evaluation of *Akanthomyces attenuatus* JEF 147 at different carbon sources.

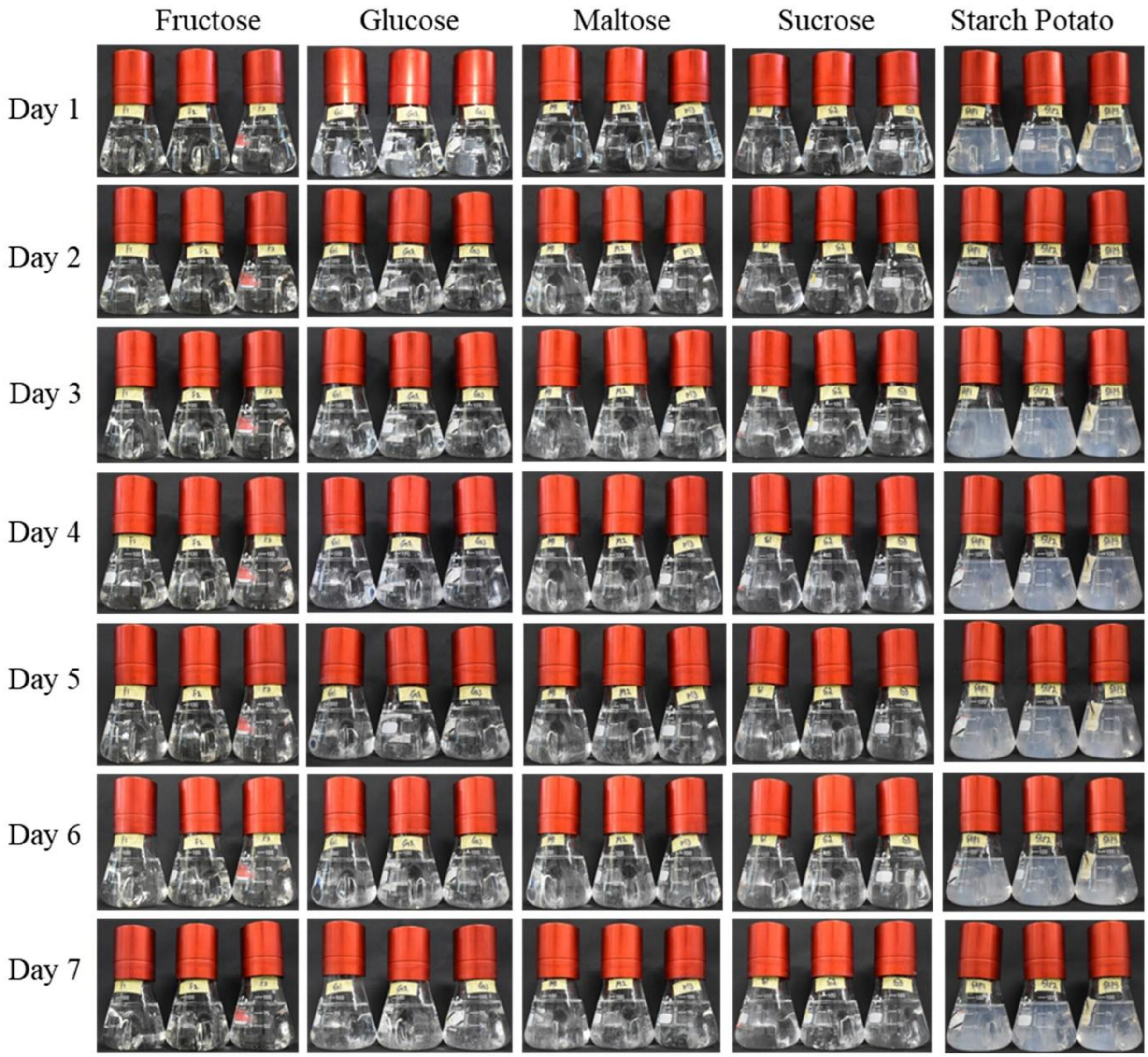

Phenotypic evaluation of *Akanthomyces attenuatus* JEF 147 isolate culture on a 1% nitrogen source.

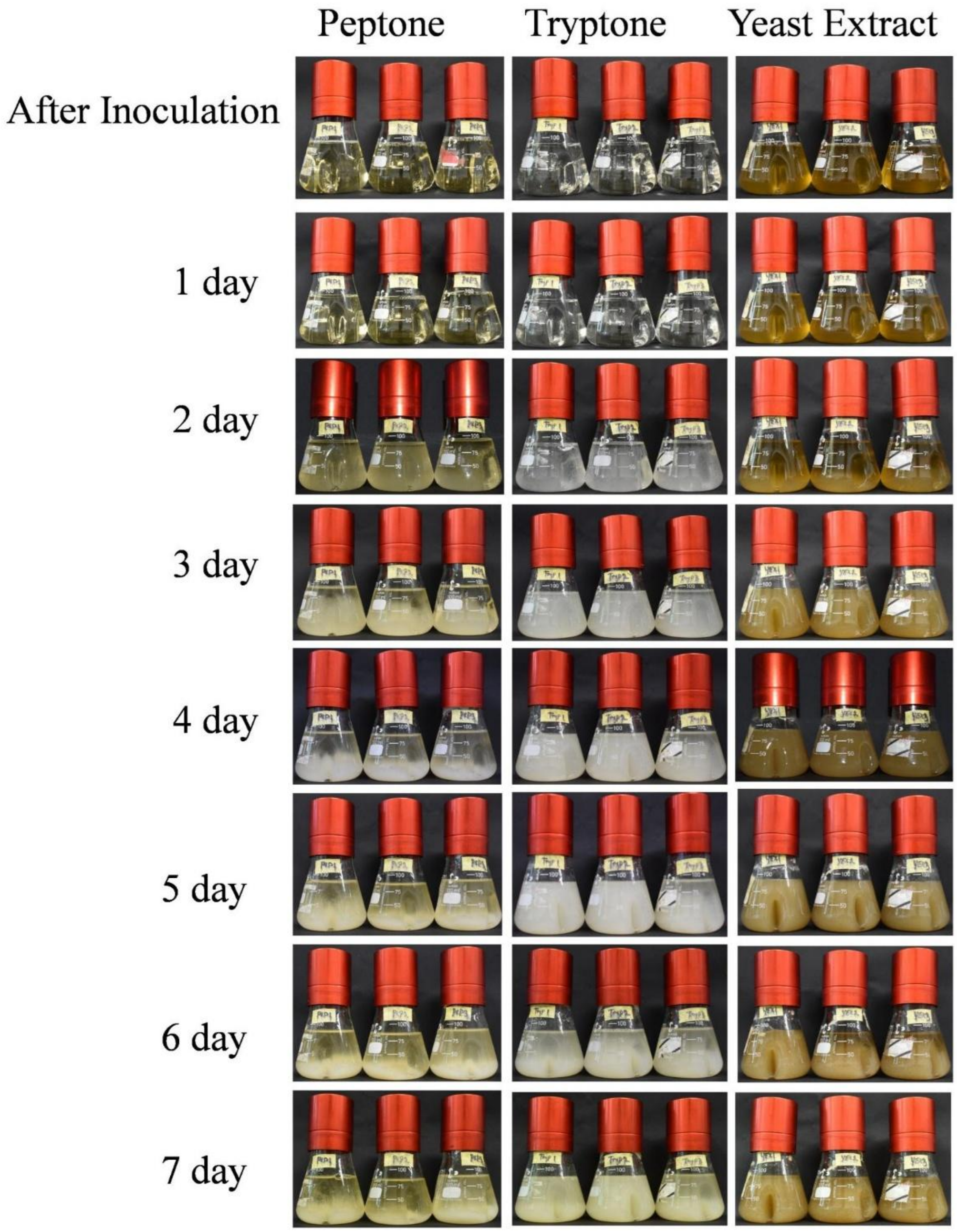

Carbon and nitrogen source combination with different mixture ratio.

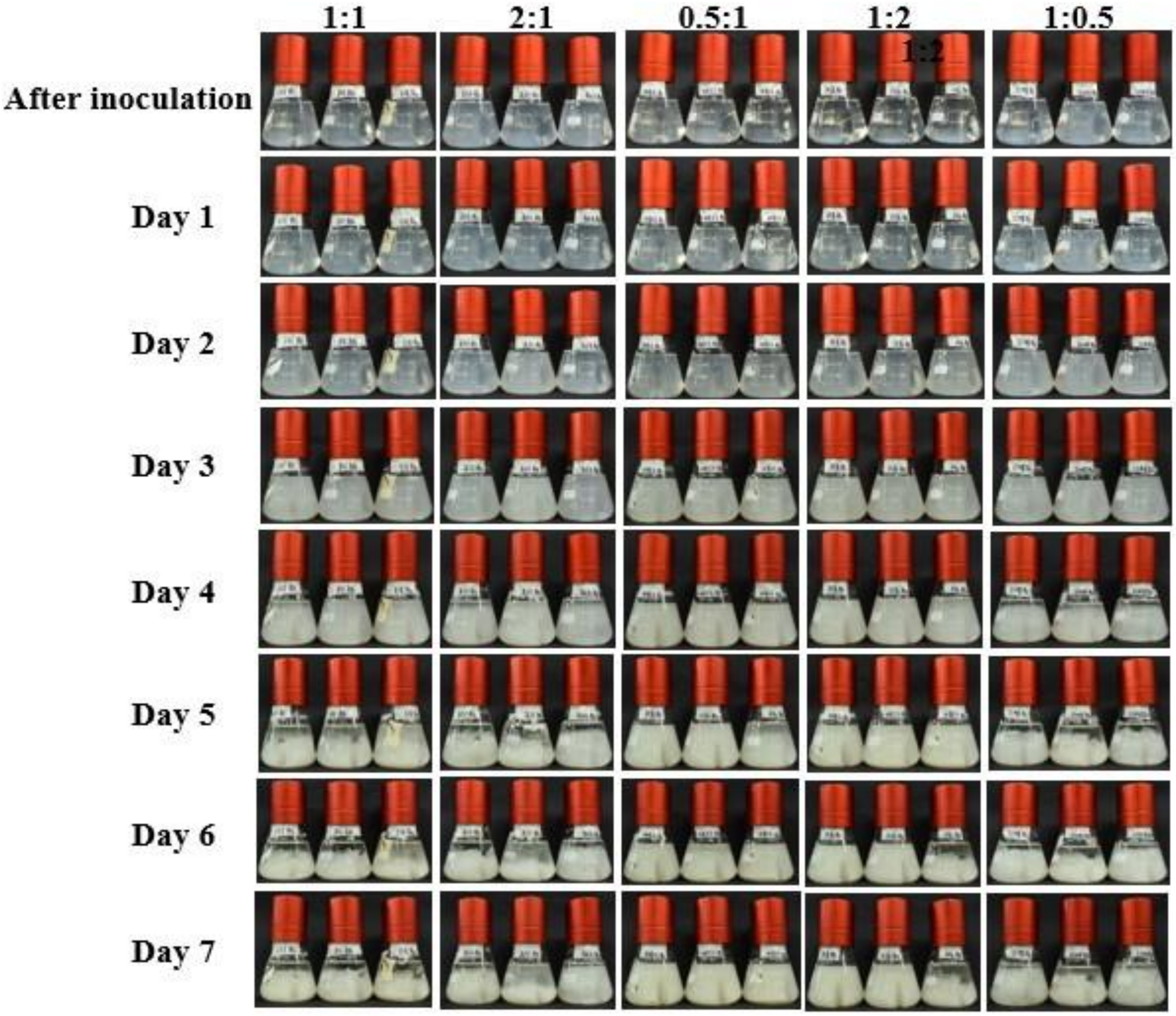

Phenotypic evaluation of *Akanthomyces attenuatus* JEF 147 in different trace metal sources with the combination of a C0.5:N1 ratio.

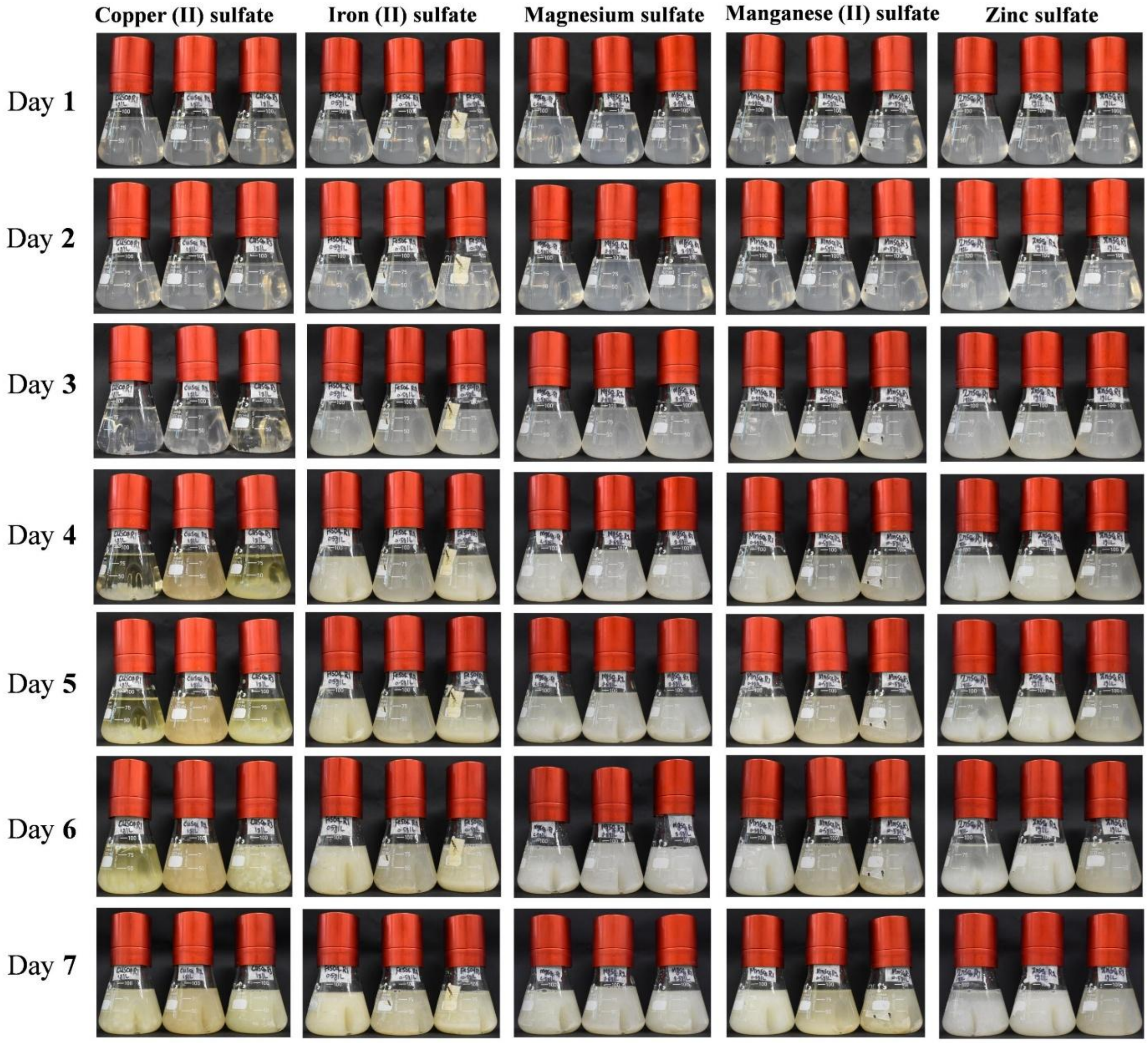

Supplement: Supplementary file 1 [file SupplementaryFile1.pdf]
